# Endometrial Proliferation and Heavy Menstrual Bleeding Associated With Continuous Oral Contraceptive Use

**DOI:** 10.7759/cureus.71450

**Published:** 2024-10-14

**Authors:** Avarie Willette, Elliot Cazes

**Affiliations:** 1 Obstetrics and Gynecology, Lake Erie College of Osteopathic Medicine, Bradenton, USA; 2 Obstetrics and Gynecology, New Tampa OBGYN, Tampa, USA

**Keywords:** continuous oral contraceptives, endometrial proliferation, heavy menstrual bleeding (hmb), hormonal birth control, oral progesterone

## Abstract

Continuous oral contraceptives are intended for three months of levonorgestrel/ethinylestradiol use followed by 10 days of low-dose ethinylestradiol. When taken for extended periods without withdrawal bleeds, side effects may include heavy menstrual bleeding and endometrial proliferation. A 41-year-old female presented to the OB-GYN clinic with two months of heavy menstrual bleeding after 10 years of Seasonique use without withdrawal bleeds (10 days of low-dose ethinylestradiol). Her endometrial thickness measured 3 mm, and luteinizing hormone and follicle-stimulating hormone levels were <0.2 mIU/mL and 0.9 mIU/mL, respectively, below the normal range for a reproductive-aged female. The patient’s heavy menstrual bleeding and abnormal lab results were attributed to long-term use of Seasonique, a continuous oral contraceptive. Treatment included Nexstellis to regulate her menstrual cycles and Aygestin to induce endometrial atrophy. This case highlights the implications of long-term continuous oral contraceptive use and the importance of adhering to medication guidelines to prevent adverse effects.

## Introduction

Pathophysiology of heavy menstrual bleeding and endometrial proliferation

The average menstrual cycle in women lasts 28 days, with four to five days of bleeding caused by the shedding of the superficial layer of the endometrium, known as the stratum functionalis. Following this shedding, the proliferation of the stratum functionalis is primarily driven by estrogen. The subsequent drop in progesterone after its mid-cycle surge triggers endometrial shedding during menstruation. In continuous oral contraceptive regimens, progesterone levels remain stable, preventing the shedding of the endometrium [1]. Prolonged use of oral contraceptives without withdrawal bleeds may lead to excessive endometrial growth [1]. However, when used as directed, there is no risk of endometrial proliferation. Hee et al. reported that in seven of eight continuous contraceptive users, the endometrium was completely inactive when the regimen was followed correctly [2]. As a result, one would expect the endometrial lining to measure 0 mm in those on continuous oral contraceptives, compared to 3-4 mm in reproductive-aged females. The definition of heavy menstrual bleeding varies among physicians and patients, but a widely accepted criterion is the need to change a tampon or pad every one to two hours.

Seasonique

Seasonique is an extended contraceptive regimen consisting of 84 days of levonorgestrel/ethinylestradiol (150/30 µg) followed by 10 days of ethinylestradiol (10 µg). When used as prescribed, this regimen allows for four withdrawal bleeds per year, compared to 13 with traditional oral contraceptives [3]. Notably, Seasonique contains no estrogen-free days.

## Case presentation

Patient presentation

A 41-year-old female presented to the OB-GYN office with a two-month history of heavy menstrual bleeding. The patient reported bleeding through up to one pad per hour on multiple days, occurring on and off for two months. She experienced menarche at age 12 and noted the following premenstrual symptoms accompanying each cycle: headache, bloating, cramps, acne, constipation, and back pain. The patient had a history of monthly menstrual cycles with irregular bleeding episodes. She was nulliparous and had never experienced a miscarriage, stillbirth, or elective abortion. For 10 years, the patient had taken Seasonique (0.15-0.03 and 0.01 mg) once daily for pregnancy prevention, as prescribed by her primary care provider. Additionally, she had a history of anxiety, which was treated with alprazolam.

Laboratory results

Laboratory results were collected (Table 1). Luteinizing hormone (LH) and follicle-stimulating hormone (FSH) levels were significantly low for a reproductive-aged female, while estradiol and progesterone were within normal limits. Both transabdominal and transvaginal scans were performed, revealing that the uterus measured 5.8 × 2.8 × 3.8 cm with no masses identified. There were no endometrial masses or abnormal fluid collections, and the endometrium measured 3 mm in thickness.

**Table 1:** Hormone panel including reference ranges FSH, follicle-stimulating hormone; LH, luteinizing hormone

Test name	Result	Reference range
LH	<0.2 mIU/mL	Follicular phase: 2.4-12.6 mIU/mL; ovulation phase: 14.0-95.6 mIU/mL; luteal phase: 1.0-11.4 mIU/mL; postmenopause: 7.7-58.5 mIU/mL
FSH	0.9 mIU/mL	Follicular phase: 3.5-12.5 mIU/mL; ovulation phase: 4.7-21.5 mIU/mL; luteal phase: 1.7-7.7 mIU/mL; postmenopause: 25.8-134 mIU/mL
Estradiol	15 pg/mL	Follicular phase: 12.4-233 pg/mL; ovulation phase: 41.0-398 pg/mL; luteal phase: 22.3-341 pg/mL; postmenopause: <5-138 pg/mL
Progesterone	0.5 ng/mL	Follicular phase: 0.057-0.893 ng/mL; ovulation phase: 0.121-12.0 ng/mL; luteal phase: 1.83-23.9 ng/mL; postmenopause: 0.03-0.126 ng/mL

Diagnosis 

After reviewing the lab results, the patient’s heavy menstrual bleeding, along with the abnormal FSH and LH levels, was attributed to her use of Seasonique oral contraceptives without withdrawal bleed intervals for 10 years. The low FSH and LH levels indicated suppressed ovarian function due to the long-term use of contraceptives in this patient.

Treatment

The patient was started on Nexstellis oral contraceptive, which involves 24 active pills containing 12.4 mg of E4, a naturally occurring estrogen, and 3 mg of drospirenone, a commonly used progestin [1]. Aygestin, a synthetic progesterone, was also added to her regimen. The patient was instructed to report any episodes of heavy bleeding; however, she did not report any continued heavy bleeding.

## Discussion

Nexstellis was chosen to regulate the patient’s menstrual cycle and prevent pregnancy. This regimen will allow for monthly withdrawal bleeds, helping to prevent the buildup of endometrial tissue. Aygestin was added to induce endometrial atrophy and address the underlying cause of her heavy menstrual bleeding. The patient’s suppressed LH and FSH values were consistent with long-term oral contraceptive use. After more than seven years of use, FSH levels have been shown to decrease by 70%, while LH values are suppressed by 20-30% [4]. This case highlights the importance of adhering to medication instructions, particularly with continuous oral contraceptive use.

## Conclusions

In this patient, long-term use of continuous oral contraceptives affected her LH and FSH levels, as well as her endometrial thickness. Without withdrawal bleeds, her endometrium was thickened compared to other reproductive-aged patients on oral contraceptives, contributing to her heavy menstrual cycles. The risks and benefits of long-term continuous oral contraceptive use should be discussed with patients before initiation. When evaluating a patient with heavy menstrual bleeding on an oral contraceptive regimen, adherence to medication instructions must be thoroughly assessed. It is also essential to consider the patient’s health literacy and tailor education to their level of understanding to ensure they are informed about their condition and treatment options. Future studies should investigate the long-term effects of continuous oral contraceptive use on heavy menstrual bleeding and endometrial hyperplasia.

## References

[1] Hapangama DK, Bulmer JN (2016). Pathophysiology of heavy menstrual bleeding. Womens Health (Lond).

[2] Hee L, Kettner LO, Vejtorp M (2013). Continuous use of oral contraceptives: an overview of effects and side-effects. Acta Obstet Gynecol Scand.

[3] Burness CB (2015). Extended-cycle levonorgestrel/ethinylestradiol and low-dose ethinylestradiol (Seasonique®): A review of its use as an oral contraceptive. Drugs.

[4] Goldzieher JW, Kleber JW, Moses LE, Rathmacher RP (1970). A cross-sectional study of plasma FSH and LH levels in women using sequential, combination or injectable steroid contraceptives over long periods of time. Contraception.

